# Editorial: Vitamin D and aging: associations with mortality, cognition, chronic diseases, and metabolic conditions in elderly individuals

**DOI:** 10.3389/fendo.2026.1830429

**Published:** 2026-04-16

**Authors:** Yupeng Li, Hongzhou Liu, Huikun Liu, Hui Tian

**Affiliations:** 1 NHC Key Laboratory of Hormones and Development (Tianjin Medical University), Tianjin Key Laboratory of Metabolic Diseases, Tianjin Medical University Chu Hsien-I Memorial Hospital & Tianjin Institute of Endocrinology, Tianjin, China; 2 The General Hospital of the Third Division of Xinjiang Production and Construction Corps, Xinjiang, China; 3 Department of Endocrinology, Aerospace Center Hospital, Beijing, China; 4 Tianjin Women’s and Children’s Health Center, Tianjin, China; 5 Department of Endocrinology, The Second Medical Center, Chinese PLA General Hospital, Beijing, China

**Keywords:** aging, bone density, cognition, metabolic syndrome, mortality, neurodegeneration, vitamin D

## Introduction

Global population aging has intensified the search for modifiable determinants of healthspan and longevity. Vitamin D, a fat-soluble secosteroid, is indispensable for calcium homeostasis, bone metabolism, and a broad array of physiological functions (1). Aging amplifies the risk of vitamin D inadequacy through reduced cutaneous synthesis, impaired renal and hepatic activation, and diminished sun exposure. A recent global meta-analysis found that approximately 59.7% (95% CI 45.9–72.1) of elderly individuals are vitamin D-deficient (<20 ng/mL) (2). Accumulating epidemiological evidence links low vitamin D status with musculoskeletal disease, cardiovascular disease, type 2 diabetes, neurocognitive dysfunction, and all-cause mortality (3). It is important to note, however, that the presumed extra-skeletal effects of vitamin D remain less well understood and documented than the well-established skeletal effects, as highlighted in the recent Consensus Statement on Vitamin D Status Assessment and Supplementation: Whys, Whens and Hows (4). This comprehensive, up-to-date consensus underscores that while the skeletal benefits of vitamin D are well characterized, the evidence for extra-skeletal effects is more limited and inconclusive, warranting careful interpretation of the observational associations presented in the literature. This Research Topic assembles nine rigorous investigations that illuminate the multifaceted relationships between vitamin D and aging across bone health, mortality risk, cognitive function, metabolic regulation, and organ-specific outcomes in elderly populations.

## Skeletal health and mortality: threshold effects and inflammatory mediators

Serum 25-hydroxyvitamin D is the best clinical indicator of vitamin D status; even mild decreases are associated with secondary hyperparathyroidism, which is associated with adverse effects on bone metabolism in the elderly. While intermittent high-dose vitamin D3 reliably raises 25(OH)D levels, associations with fracture and fall reduction remain inconsistent (5), and the DO-HEALTH trial found only modest, clinically uncertain benefits on hip bone mineral density (6). Yin et al. investigated threshold associations of bone mineral density with mortality risk, comprehensively analyzing BMI as a potential mediator in older populations. Their work demonstrates that the association between bone density and survival is nonlinear, with critical thresholds varying by body composition, providing important nuance to the clinical interpretation of densitometry results. Complementarily, He et al. demonstrated that the platelet-to-albumin ratio—a novel inflammatory-nutritional marker—associates with spinal BMD in elderly patients with thoracolumbar fragility fractures, offering a practical tool for fracture-risk stratification. Vitamin D supplementation, especially combined with physical exercise (7), is associated with improvements in muscle strength and postural balance in osteoporotic patients, and reduced fall risk (8, 9).

## Cognitive and neurodegenerative dimensions

Vitamin D3 is associated with nervous system function and has been linked to Alzheimer’s disease (AD) and Parkinson’s disease (PD); deficiency is correlated with cognitive decline, heightened neuroinflammation, and shorter leukocyte telomeres (10). Although vitamin D levels below 20 ng/mL are associated with increased dementia risk, evidence from observational studies remains inconclusive due to methodological heterogeneity (11). Li et al. found, through a centenarian cohort of Chinese women, that vitamin D status is a significant correlate of cognitive health even at extreme old age. Lv et al. used advanced neuroimaging to show that vitamin D-linked vulnerability is associated with superior frontal gyrus connectivity alterations and cognitive impairment in PD—suggesting potential mechanistic pathways of neuroprotection. Advanced metabolomic approaches have revealed that vitamin D metabolites beyond total 25(OH)D, including 24,25-dihydroxyvitamin D3 and 3-epimer-25-hydroxyvitamin D3, may provide additional insights into cognitive function, with sex-based differences in vitamin D-cognition associations potentially reflecting hormonal effects (12).

### Mortality Risk and Cardiovascular Outcomes

Among patients with established cardiovascular disease, a nonlinear inverse association exists between 25(OH)D and all-cause, cancer, and respiratory mortality; each 10 nmol/L increment is associated with a 12% lower all-cause mortality risk in deficient patients (13). UK Biobank data (409,822 participants; 15.9-year follow-up) show that regular vitamin D supplementation is associated with a lower risk of atherosclerotic cardiovascular disease (HR 0.94, 95% CI 0.90–0.98) (14). In coronary artery disease, RCT evidence is mixed for the general elderly population, though diabetic subgroups may show benefits (15). Yan et al. examined circulating vitamin D and FSH in climacteric women with menopausal syndrome, identifying hormone-nutrient interactions associated with subclinical atherosclerosis. Additionally, secondary hyperparathyroidism due to vitamin D deficiency is independently associated with more than a fourfold higher risk of carotid plaque when combined with elevated PTH (16).

## Metabolic consequences and organ-specific manifestations

The VITAL trial (n = 22,220) found that vitamin D3 at 2,000 IU/day was not associated with a significant reduction in incident type 2 diabetes overall, although a meta-analysis of four trials reported a modest benefit (HR = 0.89, 95% CI 0.80–0.99) (17, 18). Long-term supplementation (800 IU/day for 30 months) in middle-aged to elderly diabetic patients was associated with significant reductions in fasting insulin, HOMA-IR, non-HDL-cholesterol, hs-CRP, and uric acid (19). Hong et al. identified an association between serum 25(OH)D and dyslipidemia risk in elderly diabetic foot ulcer patients, with implications for wound healing and limb preservation. Miao et al. revealed important interactions between vitamin D status, sex hormones, and body composition in Chinese older adults; vitamin D2 also associates with energy-regulating hormones (insulin, adiponectin, leptin), suggesting a role in metabolic homeostasis (20). Two additional papers address organ-specific outcomes: Dai et al. identified an association between hydroxyvitamin D and pulmonary function in elderly CKD patients—a population doubly vulnerable to vitamin D deficiency and respiratory complications (21); and Liang et al. found that AMD patients had significantly lower 25(OH)D than controls (22.98 ± 7.30 vs. 26.12 ± 9.81 ng/mL, p = 0.013), with levels inversely correlated with disease stage and positively with apolipoprotein E, extending vitamin D’s relevance to age-related sensory impairment.

## Conclusion

Collectively, these nine articles advance understanding of vitamin D in aging across multiple domains. They confirm that vitamin D deficiency is prevalent and consequential in older populations, with nonlinear relationships and population-specific threshold associations suggesting that optimal ranges vary by clinical outcome and subgroup. Notably, most of these studies report associations rather than causal relationships, and the mechanistic pathways remain to be fully elucidated. Novel mechanisms—from neural connectivity to inflammatory and endocrine pathways—have been proposed to explain vitamin D’s pleiotropic associations, though these require further confirmation. Clinically actionable insights are provided for high-risk groups including diabetic foot ulcer patients, CKD patients, and women at the menopausal transition. As guest editors, we sincerely thank the contributing authors—Yin et al., He et al., Li et al., Lv et al., Yan et al., Hong et al., Miao et al., Dai et al., Liang et al., and their colleagues—as well as the reviewers and the editorial team at *Frontiers in Endocrinology*. We hope this Research Topic will catalyze further research and evidence-based recommendations to improve health outcomes for aging populations worldwide.
